# Phytochemical composition and antioxidant activities of some wild edible plants locally consumed by rural communities in northern Uganda

**DOI:** 10.3389/fnut.2023.1070031

**Published:** 2023-04-04

**Authors:** Alfred Nyero, Godwin Upoki Anywar, Innocent Achaye, Geoffrey Maxwell Malinga

**Affiliations:** ^1^Department of Chemistry, Faculty of Science, Gulu University, Gulu, Uganda; ^2^Department of Plant Sciences, Microbiology and Biotechnology, College of Natural Sciences, Makerere University, Kampala, Uganda; ^3^Department of Biology, Faculty of Science, Gulu University, Gulu, Uganda

**Keywords:** polyphenolic compounds, phenolics, methanolic extracts, *in vitro* antioxidant activity, wild leafy vegetables, radical scavenging activity

## Abstract

**Background:**

*Acalypha rhomboidea*, *Asystacia gangetica*, *Crassocephalum sacrobasis*, *Crotalaria ochroleuca*, *Heterosis rotundifolia*, *Hibiscus cannabinus*, *Hibiscus* sp., *Hibiscus surratensis*, *Ipomoea eriocarpa*, *Maerua angolensis*, *Senna obtusifolia* and *Vigna membranacea* are among the common wild edible plants in the Acholi sub-region, northern Uganda. This study evaluated the phytochemical constituents and antioxidant potential of the plants.

**Methods:**

Fresh leaves collected from each plant species were air-dried under shade. The phytochemical contents of the ethanol and petroleum ether extracts were determined using standard protocols. The antioxidant content of the methanolic extracts was assessed by 2,2-diphenyl-1-picrylhydrazyl (DPPH) assay.

**Results:**

Preliminary phytochemical analyses indicated the presence of tannins, reducing compounds, alkaloids, flavonoids, flavons aglycones, flavanosides, anthracenosides, anthocyanosides, volatile oils, coumarins, steroid glycosides, sterols and triterpenes. However, the extracts did not contain any emodols and saponins. The results of the quantitative phytochemical analysis showed that the contents of different phytochemicals detected varied significantly (*p* < 0.05) among the selected plants. The amount of tannins in mg/g (gallic acid equivalent) of dry weight varied from 3.90 ± 0.16 in *C. ochroleuca* to 10.41 ± 0.78 in *I. eriocarpa*, total flavonoids in RE, mg/g dry matter from 4.07 ± 0.11 in *I. eriocarpa* to 14.94 ± 0.08 in *S. obtusifolia.* Total alkaloids in mg/100 g ranged from 1.59 ± 0.30 in *I. eriocarpa* to 6.37 ± 0.24 in *Hibiscus* sp. Total phenolic content in GAE, mg/g dry matter ranged from 13.39 ± 0.26 in *A. rhomboidea* to 64.25 ± 0.54 in *I. eriocarpa*. The *in vitro* antioxidant assays revealed substantial free radical scavenging activity in all the plants. Antioxidant activity expressed as IC_50_ (ppm) ranged from 13.39 for *A. rhomboidea* to 64.84 for *I. eriocarpa,* compared to 12.82 for ascorbic acid standard. The total phenolic compounds and total tannins had significant and positive correlations with DPPH free radical scavenging activity.

**Conclusion:**

The findings of this study provide evidence that the species are good natural sources of phytochemicals and antioxidants, whose regular consumption could provide human health benefits by protecting against oxidative stress related diseases. Further research is needed on the structural characterization of the phytochemicals, profiling the plant extracts with high antioxidant activity and determining the antimicrobial activities.

## Introduction

Many countries worldwide, including those in sub-Saharan Africa, are experiencing increased incidences of chronic non-communicable diseases. This is partly due to unhealthy diets (1, 2). It has been established that consumption of wild edible plants can lower the incidences of some non communicable diseases (3), because they contain a variety of phytochemicals that have therapeutic potentials and can prevent many human diseases including cancer and cardiovascular diseases occurring (4, 5).

Phytochemicals are secondary plant metabolites that include phenolic compounds such as tannins, flavonoids, saponins and glycosides (6–9). Although they have no known roles in plant cell metabolism, the plants continuously synthesize phytochemicals for defense roles and to protect plants from possible environmental harm, e.g., against pathogens and herbivores attacks (10, 11). Many phytochemicals have proven human health benefits (12). For instance, phenolic compounds are of great importance due to their potent antioxidant or free radical scavenging activities (13, 14), antiseptic properties (15) and anti-inflammatory roles (16, 17). Alkaloids are powerful drugs with anti-inflammatory, antimalarial, antimicrobial and antispasmodic activities. Similarly, phytosteroids are known to have cardiotonic, antibacterial (18) and mixed agonistic/antagonistic activity to animal steroid receptors (19). Anthocyanins are flavonoid compounds that help the immune system to work more efficiently to protect against viral infections (20, 21). The phenolic compounds display antioxidant activities, which are important for healthy functions in the cells of both plants (22), and humans (23, 24).

Antioxidants are free radical scavengers that act by donating electrons to the electron-deficient free radicals, thus, rendering the radicals harmless (25–27). These free radicals include reactive oxygen species (ROS) such as superoxide anion radical and hydroxyl radical, hydrogen peroxide and singlet oxygen formed from metabolism (28) and reactive nitrogen species such as nitric oxide and peroxynitrite. Metabolic reaction in the cell mitochondria is the body’s main source of free radicals (29, 30). Exogenous sources of free radicals include exposure to chemical contaminants in food, charred food, cosmic radiation from space and smoking (31). A myriad of metabolic reactions in the body involves synthesizing various chemical intermediates susceptible to free radical attacks (32, 33). Free radicals’ interference in the body reactions alters amino acid configurations, denaturing enzymes. Free radicals attack nucleic acids, breaking down the nucleotide strands when they occur at the sugar linkages. Free radicals can also play helpful roles in some instances, such as apoptosis (34). For healthy body functions, there must be a balance between generating and removing free radicals. Many oxidative stress-related diseases, such as heart diseases and cancer (35, 36) result from the accumulation of free radicals in the body. Therefore, removing or scavenging free radicals significantly reduces the oxidative stress or imbalance between the free radicals generation and their removal in the body. Moderate amounts of ROS play an essential role in cell signaling involving apoptosis and gene expression and can serve as both intra- and inter-cellular messengers (37–39).

Several wild edible plant species are utilized by the Acholi communities in northern Uganda (40). Despite the abundance and diversity of these plants, many of them have not been explored for their phytochemical and antioxidant properties (41). The phytochemical and antioxidant screening of these plants is a important for verification of their continued consumption and future utilization as health-promoting foods. Therefore, this study aimed to analyse; (i) the phytochemical constituents, (ii) *in vitro* antioxidant potential of extracts of selected wild edible plant species consumed by the Acholi communities in northern Uganda, and (iii) to correlate the phytochemical contents with the antioxidant activities.

## Materials and methods

### Collection of plant materials

**Figure 1:**
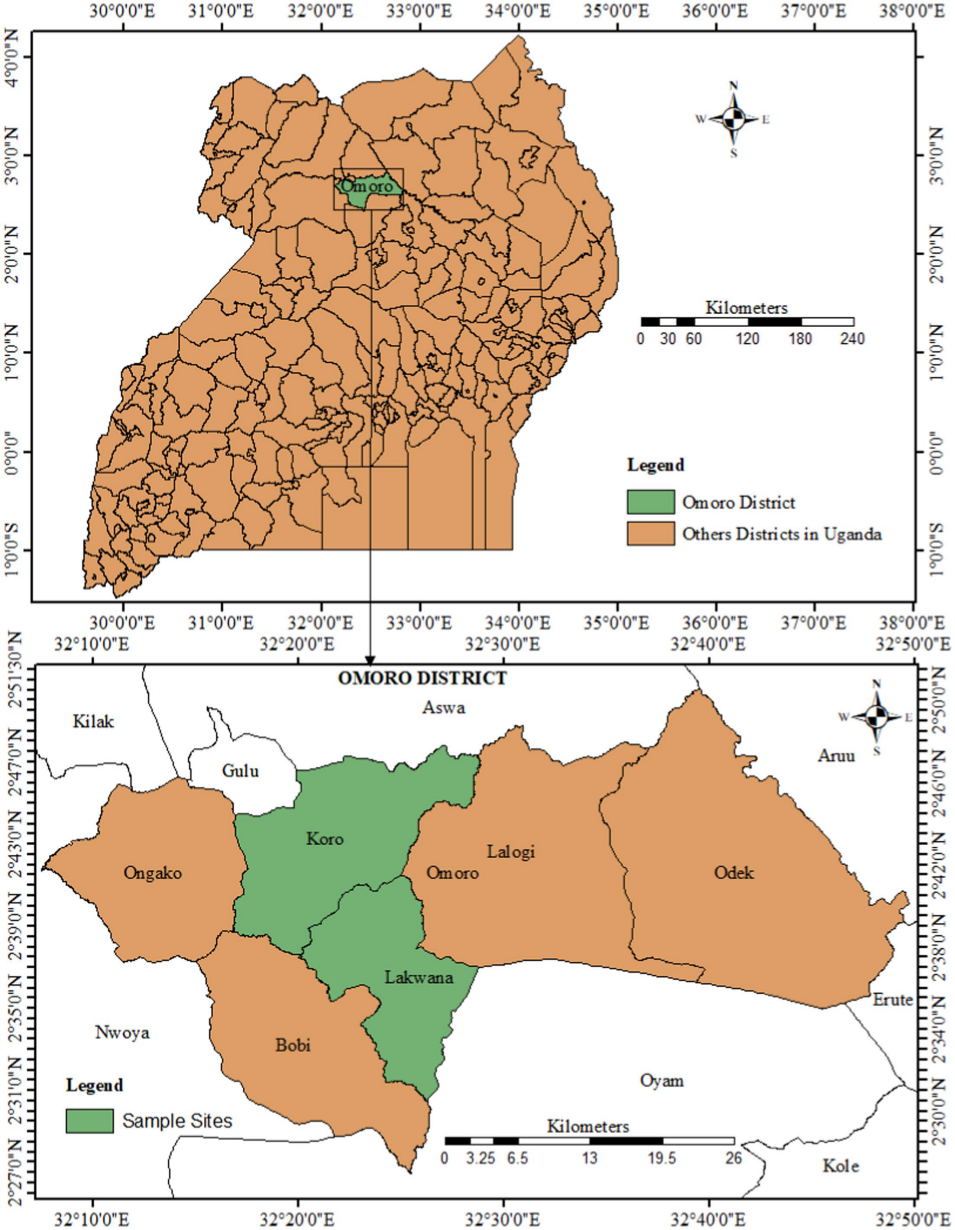
Location of study areas in Omoro district, northern Uganda. The map was created by the authors using ArcGIS version 10.3.1.

Fresh leaves (about 1 kg) of each selected wild edible plants, named as *Acalypha rhomboidea* Raf., *Asystacia gangetica* (L.) T. Anderson, *Crassocephalum sacrobasis* (DC.) S. Moore, *Crotalaria ochroleuca* G. Don., *Heterotis rotundifolia* (Sm.) Triana, *Hibiscus cannabinus* L., *Hibiscus* sp., *Hibiscus surratensis* L., *Ipomoea eriocarpa* R.Br., *Maerua angolensis* DC., *Senna obtusifolia* (L.) Irwin & Barneby and *Vigna membranacea* A. Rich., were collected at the flowering stage following the standard guidelines (42) for plant sample collection from locations in Omoro district in northern Uganda (Figure 1) between November 2019 and March 2020. Based on a previous record of Ugandan wild edible plants by Goode (43), these plants are used as a vegetable to accompany staple foods during periods of food scarcity. *Acalypha rhomboidea* is an annual garden weed used during food scarcity around April–May. *Asystica gangetica* (Ladyelcol), family Acanthaceae is a native wild-leafy vegetable that mostly grow in forest habitats. It grows up to 1 m high with extensive branching during the wet season. *Crassocephalum sacrobasis* is an annual weed of the garden used around April–May. *Crotolaria ochrolenca* (locally called Lawija) is an erect much-branched or short-lived leaf biennial vegetable herb growing up to 2.5 m tall. *Heterosis rotundifolia* (locally called Odwanga/Cunbit) is a herbaceous flowering plant found in wetlands and riverbanks. *Hibiscus cannabinus* (Lagoroto in Acholi) from the family Malvaceae is an annual herbaceous dicotyledonous wild plant. The shoots or young leaves and sometimes the flowers and young fruits are used as vegetables during the wet season. *Hibiscus surratensis* (Gwanya in Acholi), belongs to the family Malvaceae and grows in bushes around wetlands. *Hibiscus* sp. (Nyarogenga in Acholi) is an annual herb in the Malvaceae family which grows up to 1.5 m high. *Ipomoea eriocarpa* (Padowiakuri), from the family Convolulaceae, is an annual crawling herb traditionally used as a leaf vegetable in Uganda. *Maerua angolensis* (locally called Odwee) is a shrub or small tree usually growing up to 4 m high in savannah and galleried forest areas of tropical Africa. The leaves and tender parts are eaten as a vegetable in times of food shortage during the dry season. *Senna obtusifolia*, locally called Oyado in Acholi. It is an erect and bushy biennial or short-lived perennial herb in the family Fabaceae growing to a height of 1.5–2.5 m. The leafy vegetable is an important source of food for rural populations during the wet season. *Vigna membranacea* (locally called Boo Ayom) is a climbing cow-pea like plant/vine common on cultivated land and bushland. The leaves are used as vegetable during the rainy season, March–May and November–January. The plant samples were transported in clean polythene bags to the laboratory. Taxonomic identification of these plant species was made by a plant taxonomist Mr. Rwambindore Protease at the Makerere University Herbarium in a previous study Nyero et al. (40).

### Sample preparation and extraction

The leaves were removed and gently washed to remove any debris, air-dried under shade at room temperature for 5 to 10 days in the laboratory. The dried plant samples were ground to a fine powder using an electric grinder, sieved and packed in a polyethylene plastic bag wrapped with aluminum foil. One hundred grams of powder from each plant material plant samples were extracted using 500 ml of general-purpose grade petroleum ether (40°C) in an extraction flask with periodic stirring for 3 days at room temperature (20–25°C). The mixture was filtered over Whatman No.1 paper using Büchner funnel. The residue was dried in the fume hood until the smell of petroleum ether was removed. The dried residue was soaked in 70% ethanol, stirred for 30 min and left for 24 h with periodic shaking (44, 45). Then the extracts were filtered using Whatman No. 1 filter paper and the filtrate evaporated to dryness under vacuum using a rotary evaporator at 60°C. The crude extract was stored in a refrigerator below 4°C for subsequent analysis.

### Phytochemical screening of the plant extracts

Phytochemical screening of the crude petroleum ether and ethanol extracts of all plants for the presence or absence of tannins, saponins, reducing compounds, alkaloids, flavonoids, flavons aglycones, flavanosides, anthracenosides, anthocyanosides, volatile oils, courmarins, emodols, steroid glycosides, sterols and triterpenes was carried out using standard methods (7, 46–49) with minor modifications.

## Quantification of phytochemicals

### Determination of total tannin contents

Total tannins were determined using the Folin–Ciocalteu method (50) with minor modifications. Plant powder (0.1 g), from each sample was extracted in 10 ml of distilled water. In each case, the extract (0.1 ml) was added to a volumetric flask (10 ml) containing 7.5 ml of distilled water. A drop of Folin–Ciocalteu reagent (0.5 ml) and 35% sodium carbonate (1 ml) solution were added. The mixture was diluted to 10 ml with distilled water, shaken and kept in the dark at room temperature for 30 min. Absorbances for the test and standard solutions were measured against distilled water as a blank at 725 nm. The regression equation *Y* = 0.39X + 0.023; *R*^2^ = 0.999 obtained from the gallic acid standard curve (0, 10, 20, 40, and 50) μg/ml was used to express the results as mg equivalents of gallic acid, GAE/g.


$$C=C_{1}\frac{v}{m}$$

C = total tannin in terms of gallic acid equivalent (GAE) mg/g, *C*_1_ = concentration of gallic acid derived from the standard curve, *v* = volume of extract in milliliters, and *m* = weight of plant extract in grams.

### Determination of total phenolic content (TPC)

The total phenolic content (TPC) in each extract was determined using the Folin–Ciocalteau method (51), with minor modifications. Gallic acid was used as a standard. The plant powder (0.1 g) was extracted in 10 ml of distilled water for each sample. Then 0.5 ml of Folin–Ciocalteau reagent was added to 0.1 ml of each plant extract solution. The total volume of the extract was reconstituted to 8.5 ml with distilled water, shaken and the resulting mixture was kept at room temperature in the dark for 10 min. Then 20% sodium carbonate (1.5 ml) solution was added, mixed thoroughly. The solution was allowed to stand in a water bath at 40°C for 20 min. Finally, the absorbance was measured at 755 nm using a UV-spectrophotometer. The gallic acid standard curve *Y* = 0.014*X* + 0.036; *R*^2^ = 0.997 was obtained using dilutions (0, 10, 20, 40, and 50 μg/ml) for measuring the TPC, which was expressed as mg of gallic acid equivalent per gram (mg GAE/g) of dry sample. TPC was determined and expressed as mg of gallic acid equivalent per gram (mg GAE/g) of dry sample (52).

### Estimation of total flavonoid content (TFC)

The total flavonoid content (TFC) in each sample was determined using the Aluminium Chloride colorimetry method described by Ordonez et al. (53). Rutin was used as a standard and TFC was determined in milligrams of rutin equivalent (mg RE/g dry weight of extract). The calibration curve *Y* = 0.10*X* + 0.018; *R*^2^ = 0.991 for rutin was obtained using different dilutions (0, 10, 20, 40, and 50 μg/ml) prepared in distilled water. Each plant powder (0.1 g) was extracted in 10 ml of 80% methanol. To 0.1 ml of the extract or standard, 0.5 ml of fresh aluminium chloride (AlCl_3_, 2%) in ethanol was added. After 1 h at room temperature, the absorbance of the reaction mixture was measured at 420 nm using a UV–visible spectrophotometer. All results were recorded from triplicate samples.

### Alkaloids content determination

Total alkaloid content was evaluated gravimetrically using a standard method (54). Five grams of the powdered sample of each plant was weighed into a 200 ml of 10% acetic acid in ethanol and allowed to stand for 4 h. The filtered extract was concentrated using a water bath at 55°C to one-quarter of the original volume. Concentrated ammonium hydroxide (NH_4_OH) was added dropwise into the extract until precipitation was complete. The whole solution was allowed to settle and the precipitate collected was washed with dilute NH_4_OH solution and then filtered. The crude alkaloid residue was weighed and calculated according to the equation: Amount of alkaloid (mg/g) = weight of precipitate/weight of the sample (55).

### Antioxidant assay

The antioxidant activity was determined using the 2,2-diphenyl-1-picrylhydrazyl (DPPH) radical scavenging assay with ascorbic acid standard according to the method described by Abdul-Wahab et al. (56) with minor modifications. Each plant powder sample (0.1 g) was cold extracted with 10 ml of methanol (99%) for 3 h by soaking, shaking intermittently and later filtered. The volume of the extract solutions was adjusted to 10 ml with methanol to make sample solutions. The sample solutions (100, 200, 300, 400, and 500 μl) and 5 ml of 0.0039% DPPH were rapidly mixed in the test tubes. After vigorous shaking, the reaction mixture was incubated in the dark at 37°C for 30 min and the absorbance was measured at 517 nm against a blank using a UV–Vis. Spectrophotometer. The ascorbic acid standard curve (*Y* = 1.468*X* + 31.17; *R*^2^ = 0.993) made from serial concentrations (5, 10, 15, and 20 μg/ml) was used as a positive control. The DPPH free radicals scavenging activity of the plant extract was expressed in terms of the effective concentration in parts per million of ascorbic acid standard and samples required to scavenge 50% of DPPH free radicals *in vitro*, IC_50_; using the equation:


$$\mathrm{DPPH free radical scavenging activity}\;\left(\%\right)=\left(\frac{A_{c}-A_{s}}{A_{c}}\right)\times 100$$

Where A_c_ = Absorbance of the blank or control solution (i.e., the absorbance of DPPH + methanol); As is the absorbance of DPPH radical + sample (i.e., the absorbance of extract or standard). The 1C_50_ values were determined from the plotted graph of the percentage of scavenging activity against the concentration of different extracts from the three replicates. A lower IC_50_ value implies a higher antioxidant activity of the sample.

### Statistical analyses

One-way ANOVA was performed to determine the significant differences between means. A *p* value < 0.05 was considered statistically significant. Then Tukey’s HSD *post hoc* comparison test was done. Linear regression analysis between the % inhibition of DPPH and the concentration was done for each sample (*n* = 3) using a linear function. The Pearson’s correlation coefficient was performed to assess the association of total phenolic, flavonoid, alkaloid and tannin contents and antioxidant activity. The statistical analysis was conducted using SPSS programme (IBM SPSS statistics version 26) for windows software. The results were presented as mean values ± SD (standard deviation).

## Results

### Phytochemical constituents

Preliminary phytochemical screening of petroleum ether and ethanol extracts from the 12 wild edible plants revealed the presence of a wide range of phytochemical compounds including volatile oils, basic alkaloids, and tannins, reducing compounds, coumarins, flavone aglycones, flavanoside, steroid glycoside, sterols and triterpenes, others occurring in higher abundance is some of the extracts as shown in Table 1. Anthracenosides were only present in two sample extracts: *H. surratensis* and *I. eriocarpa*. Anthocyanosides were found in the extracts of *H. surratensis*, *H. cannabinus*, *S. obtusifolia*, *Hibiscus* sp. and *I. eriocarpa*. None of the extracts contained saponins and emodol (Table 1).

**Table 1:** Phytochemical screening of crude extracts of 12 edible wild plant species used locally by the Acholi communities of northern Uganda.

Sample	AR	HS	CO	VM	HC	SO	AG	H	HR	IE	CR	MA
Volatile oils	+	+	+	+	+	+	+	+	+	+	+	+
Basic alkaloids	+	+	+	+	+	+	+	+	+	+	+	+
Sterols and triterpenes	+	+	+	+	+	+	+	+	+	+	+	+
Emodols	−	−	−	−	−	−	−	−	−	−	−	−
Coumarins	+	++	+	++	++	++	+	++	+	++	++	+
flavone aglycones	++	++	+	++	++	++	+	++	++	++	++	++
Saponins	−	−	−	−	−	−	−	−	−	−	−	−
Tannins	+	+	+	+	+	+	+	+	+	+	+	+
Reducing compounds	+	+	+	+	+	+	+	+	++	+	++	+
Alkaloid salts	+	+	+	+	+	+	+	+	+	+	+	+
Anthracenosides	−	+	−	−	−	−	−	−	−	+	−	−
Anthocyanosides	−	+	−	−	+	+	−	+	−	+	−	−
Flavonosides	++	++	+	++	++	++	++	++	++	++	++	++
Steroid glycosides	+	+	+	+	+	+	+	+	+	+	+	+

AR = *Acalypha rhomboidea* Raf., AG = *Asystasia gangetica* (L.) T. Anderson, CR = *Crassocephalum rubens* var. *sarcobasis (Bojer ex DC.)* C. Jeffrey & Beentje, CO = *Crotalaria ochroleuca G.Don.*, HR = *Heterotis rotundifolia (Sm.) Jacq.-Fél.*, HC = *Hibiscus cannabinus L.*, HS = *Hibiscus surattensis L.*, H = *Hibiscus* sp., IE = *Ipomoea eriocarpa R. Br.*, MA = *Maerua angolensis*, SO = *Senna obtusifolia* (L.) Irwin & Barneby., and VM = *Vigna membranacea A. Rich.*; (− absence, + presence, ++ present in large amount based on color intensity from the test outcome).

Statistical analysis results of detected quantitative phytochemical contents of the 12 selected plants indicated that they were significantly different (*p* < 0.001; Table 2). As shown in Table 1, the total tannin content ranged from of 3.90 ± 0.16 in *C. ochroleuca* to of 10.41 ± 0.78 GAE, mg/g in *I. eriocarpa.* Total flavonoids varied from 4.07 ± 0.11 to 14.94 ± 0.08 RE, mg/g. The highest amount of flavonoids (14.94 ± 0.08 mg RE/g dry weight) was observed in extracts of *S. obtusifolia* while the lowest amount (4.07 ± 0.11) was detected in *I. eriocarpa.* The total phenolic content in all the plant species ranged from 13.39 ± 0.26 to 64.25 ± 0.54 mg GAE/g dry matter. *Ipomoea eriocarpa* exhibited the highest amount of TPC with 64.25 ± 0.54 GAE/g dry matter, meanwhile *A. rhomboidea* had the lowest (13.39 ± 0.26). Total alkaloids (g/100 g) varied from 1.59 ± 0.30 to *I. eriocarpa* to 6.37 ± 0.24 in *Hibiscus* sp. (Table 2).

**Table 2:** Total tannins, total flavonoids, total phenolic compounds, alkaloids and antioxidant activity of methanol extracts of 12 selected wild edible plants from the Acholi sub-region.

Sample	Total tannins (GAE, mg/g)	Total flavonoids (RE, mg/g)	Total phenolic compounds (GAE, mg/g)	Total alkaloids (g/100 g)	Antioxidant activity (IC_50_)
*Asystacia gangetica*	3.95 ± 0.06^a^	7.66 ± 0.20^d^	14.18 ± 0.63^a^	2.75 ± 0.08^bc^	14.18
*Acalypha rhomboidea*	7.30 ± 0.54^b^	5.17 ± 0.07^b^	13.39 ± 0.26^a^	1.95 ± 0.22^ab^	13.39
*Crassocephalum sacrobasis*	7.50 ± 0.33^b^	6.48 ± 0.20^c^	32.54 ± 1.33^d^	4.02 ± 0.40^d^	32.54
*Crotalaria ochroleuca*	3.90 ± 0.16^a^	11.00 ± 0.09^f^	13.72 ± 0.27^a^	4.92 ± 0.29^e^	13.72
*Heterotis rotundifolia*	4.83 ± 0.36^d^	6.69 ± 0.16^c^	45.02 ± 0.16^e^	5.67 ± 0.49^ef^	45.02
*Hibiscus* sp.	7.58 ± 0.27^b^	12.35 ± 0.22^g^	33.30 ± 0.69^d^	6.37 ± 0.24^f^	33.30
*Hibiscus cannabinus*	7.58 ± 0.33^b^	14.13 ± 0.09^h^	25.97 ± 0.36^c^	5.04 ± 0.17^e^	25.97
*Hibiscus surratensis*	3.96 ± 0.40^a^	12.83 ± 0.57^g^	28.15 ± 1.49^c^	2.53 ± 0.23^bc^	28.15
*Ipomoea eriocarpa*	10.41 ± 0.78^d^	4.07 ± 0.11^a^	64.25 ± 0.54^f^	1.59 ± 0.30^a^	64.84
*Maerua angolensis*	4.81 ± 0.28^a^	10.20 ± 0.14^e^	17.04 ± 0.35^b^	2.25 ± 0.25^abc^	17.04
*Senna obtusifolia*	8.95 ± 0.15^c^	14.94 ± 0.08^i^	27.93 ± 1.22^c^	2.83 ± 0.31^c^	27.93
*Vigna membranacea*	4.06 ± 0.43^a^	4.67 ± 0.18^ab^	18.89 ± 0.50^b^	2.82 ± 0.20^c^	18.89
Mean square	15.2	44.6	673.0	7.5	
*F*-statistics at *df* = (1,11) (*p* < 0.001)	103.3	946.9	1106.1	93.68	

GAE, mg/g: milligram of gallic acid equivalents per gram of dry residue, RE, mg/g: rutin equivalent per gram residue, IC_50_ of ascorbic acid = 12.82 ppm. Values are expressed as means ± SD (*n* = 3). Means in columns followed by different letters indicate significant difference between different plant species at the *p* < 0.05 levels by Tukey HSD *post hoc* test.

### 2,2-Diphenyl-1-picrylhydrazyl radical scavenging activity

Among the 12 plants investigated, *A. rhomboidea* exhibited the strongest free radical scavenging ability and may be used as a potential source of natural antioxidants against free radical-associated diseases. The DPPH radical scavenging of the plants determined as IC_50_ (ppm) ranged from 13.39 to 64.84 compared to 12.82 for the standard ascorbic acid used (Table 2).

### Correlation of antioxidant activity with the phytochemical contents

The results of Pearson correlation revealed a significant and positive correlation of antioxidant or DPPH radical scavenging activity (IC_50_) with total tannins (*r* = 0.600, *p* = 0.039) as well as total phenolics (*r* = 0.999, *p* < 0.001). However, there were no significant correlations with total flavonoids (*r* = −0.238, *p* = 0.457) and total alkaloid contents (*r* = 0.052, *p* = 0.873).

## Discussion

The results show that the leaves of wild edible plants consumed by the Acholi communities in northern Uganda contain a wide range of phytochemicals. All the 12 wild edible plants contained some tannins. The tannin content ranged from 3.9 to 10.4 mg/100 g. These values are higher than those in similar studies with comparable methods; for instance, Senguttuvan et al. (57) reported the tannins content of the dry powder of wild edible plants collected from Nilgiris, the Western Ghats, Tamil Nadu, India to range from 0.03 and 1.62 mg GAE/100 g. Emmanuel et al. (58) reported lower tannin content in wild edible plants from Iringa district, Tanzania, ranging from 1.05 to 19.02 mg/100 g. The observed differences could be explained by differences in geographical distribution and variability of soil nutrients (59, 60). Although tannins have traditionally been regarded as anti-nutritional factors in foods (61), they are also known to have anti-inflammatory, wound healing (62) and antibacterial properties (63) and have remarkable ability in prevention of cancer (64, 65). Therefore, wild edible plant species containing this compound may be a potential bioactive compound source in cancer treatment.

The flavonoid content of the plant species varied from 4.07 ± 0.11 in *I. eriocarpa* to 14.94 ± 0.08 RE, mg/g in *S. obtusifolia*. The total flavonoid contents in this study is similar to those reported for similar species in previous works (66) on selected Ugandan medicinal plant foods, i.e., 12.0 ± 0.2 for *S. obtusifolia* and 12.9 ± 1.0 for *Hibiscus* sp. In contrast, other studies have shown higher flavonoid content of wild edible plants compared to ours. For example, Lamien-Meda et al. (67) showed the flavonoids content of 14 wild edible fruits of Burkina Faso to vary from 1.70 ± 0.35 to 116.05 ± 3.04 RE, mg/g. Andabati and Muyonga (66) studied selected Ugandan traditional medicinal foods, and indicated higher flavonoid content in *H. cannabinus* (38.4 ± 0.9 RE, mg/g) and *I. eriocarpa* (78.9 ± 2.7) than in the present study. Yang et al. (68) recorded lower total flavonoids contents of 2.54 mg/g fresh weight in wild edible plants of the World Vegetable Center-southern Taiwan. Flavonoids confer characteristic tastes in foods, thus promoting peculiar tastes in prepared foods. Naturally, flavonoids are reported to have a wide range of beneficial effects on humans and are therapeutically potent against a wide range of diseases (69, 70). They exhibit their actions through effects on membrane permeability and by interfering in enzyme activity and scavenging free radicals (71, 72).

The content of total phenolic compounds in this study was high and ranged from of 13.39 ± 0.26 in *A. rhomboidea* to of 64.25 ± 0.54 mg GAE/g dry matter in *I. eriocarpa*. A previous study by Andabati and Muyonga (66) has also reported higher amount (91.9 mg GAE/g) of phenolic compounds in *I. eriocarpa.* The obtained results corresponded well with the range of the values previously reported in some other studies (e.g., (67)). According to Lamien-Meda et al. (67), the total phenolic content of the methanol extracts from some wild edible plants from Burkina Faso varied from 1.91 to 49.47 GAE, mg/g, which is comparable to those in the present study. Meanwhile, our study recorded total phenolic content lower than that of Yang et al. (68) on Argentinean wild grapefruits (320 GAE, mg/g) and Chilian variety of 117 GAE, mg/g. The variation of phytochemical contents in the wild edible plants could be attributed to the difference in plant species, environmental conditions (73), plant parts used (74) and the solvent used for extraction (75). Besides, polyphenolic compounds are a key component of all the plant-derived foods and these act primarily as antioxidants, and ant-inflammatory agents (62). Phenolic substances also have antiseptic properties (15).

The alkaloids content varied from 1.59–6.37 ± 0.24 g/100 g and it differed significantly among the wild edible plants. Some vegetables like *C. ochroleuca* are bitter due to the presence of alkaloids belonging to the quinolizidines group (76). Some alkaloids possess medicinal properties such as amoebicidal and antitumor activity (77, 78).

The results of DPPH scavenging activity assay indicate that these wild edible plants are potentially good sources of natural antioxidants. In this study, the free radical scavenging activity determined by DPPH varied from13.39 to 64.84 μg/ml, which was comparable to values (0.1 to 57.8) obtained in selected Ugandan traditional medicinal foods from Kamuli to Gulu districts (66). For example, the antioxidant activities of *H. cannabinus* and *C. ochroleuca* were 22.2 ± 1.8 and 8.8 ± 0.7 milligram ascorbic acid equivalents per gram dry weight (mg VCE gDW^−1^), compared to 25.97 and 13.72 μg/ml in this study. However, the antioxidant potential of the 12 wild edible plants is not as effective as DPPH radical scavengers when compared to ascorbic acid. This is contrary to other findings that reported a higher antioxidant capacity of plant extracts than ascorbic acid (79, 80). The low oxidative potential could be attributed to the extraction solvents used for each study (81, 82). Nonetheless, our results are comparable to the values of DPPH radicals scavenging activities of Brazilian wild medicinal plants (10 ppm), as reported by Brighente et al. (83); which was also lower than that of the ascorbic acid standard (8.4 ppm). This variation in antioxidant activities could be attributed to differences in the wild edible plant species studied, climatic conditions and soil types (84). However, one major limitation of this study was that the antioxidant activity was described by only one method, yet, antioxidant activity can be influenced by many factors and because of the complex nature of many plants and differences in the mechanism of action of antioxidants (85). Nevertheless, the highly significant and positive correlation of the DPPH radical scavenging IC_50_ with total tannins and total phenolic content could imply that tannins and phenols are the main contributors to the radical scavenging activity of the selected plants. A high correlation between the content of phenolic compounds and the scavenging effect of DPPH has also been previously demonstrated by Noreen et al. (86) in study from Pakistan and by Andabati and Muyoinga (66) in wild edible plants of Gulu and Kamuli districts in Uganda. Several factors might influence antioxidant activity and, therefore might not be fully described by a single assay. A reliable antioxidant evaluation protocol could have required employing different antioxidant activity assessment methods, such as nitric oxide radical scavenging, ferric reducing power and total antioxidant assays. Given that only one method was employed in this study, this can be a limitation of the study.

## Conclusion

This study shows that the leaves of wild edible plants consumed by the Acholi communities in northern Uganda contain a wide range of bioactive phytochemicals and are a rich natural source of antioxidants. The highest contents of both total phenolic and tannins were found in *I. eriocarpa*. *Senna obtusifolia* and *Hibiscus* sp. presented the highest contents of total flavonoid and total. Among the selected edible plants, *A. rhomboidea* provided the highest activity for antioxidants. Hence their regular consumption could provide human health benefits by protecting against oxidative stress related diseases. The high correlations confirm the roles of phenol and tannin compounds as the main contributor to the antioxidant activities of these plants. Further investigations are needed to characterize the phytochemicals in the wild edible plants, profiling the plant extracts with high antioxidant activity by LC–MS and determining their microbial activities. This will stimulate interest in wild edible plant use in the nutraceutical industries and new drug development.

## Data availability statement

The raw data supporting the conclusions of this article will be made available by the authors, without undue reservation.

## Author contributions

AN, GM, and GA designed this study. AN collected, analyzed samples, and wrote the initial draft of the manuscript. GM, IA and GA were responsible for data interpretation and editing of the manuscript. All authors contributed to the article and approved the submitted version.

## Funding

This study was funded by the African Development Bank through a scholarship grant awarded to AN.

## Conflict of interest

The authors declare that the research was conducted in the absence of any commercial or financial relationships that could be construed as a potential conflict of interest.

## Publisher’s note

All claims expressed in this article are solely those of the authors and do not necessarily represent those of their affiliated organizations, or those of the publisher, the editors and the reviewers. Any product that may be evaluated in this article, or claim that may be made by its manufacturer, is not guaranteed or endorsed by the publisher.

## References

[1] Branca F Lartey A Oenema S Aguayo V Stordalen GA Richardson R. Transforming the food system to fight non-communicable diseases. BMJ. (2019) 364:l296. doi: 10.1136/bmj.l296 30692128 PMC6349221

[2] Qiao J Lin X Wu Y Huang X Pan X Xu J. Global burden of non-communicable diseases attributable to dietary risks in 1990–2019. J Hum Nutr Diet. (2022) 35:202–13. doi: 10.1111/jhn.12904, PMID: 33834556

[3] Lyons G Dean G Tongaiaba R Halavatau S Nakabuta K Lonalona M. Macro-and micronutrients from traditional food plants could improve nutrition and reduce non-communicable diseases of islanders on atolls in the South Pacific. Plan Theory. (2020) 9:942. doi: 10.3390/plants9080942, PMID: 32722347 PMC7464995

[4] De Carvalho APA Conte-Junior CA. Health benefits of phytochemicals from Brazilian native foods and plants: antioxidant, antimicrobial, anti-cancer, and risk factors of metabolic/endocrine disorders control. Trends Food Sci Technol. (2021) 111:534–48. doi: 10.1016/j.tifs.2021.03.006

[5] Moghaddam RH Samimi Z Moradi SZ Little PJ Xu S Farzaei MH. Naringenin and naringin in cardiovascular disease prevention: a preclinical review. Eur J Pharmacol. (2020) 887:173535. doi: 10.1016/j.ejphar.2020.173535, PMID: 32910944

[6] Ashraf MA Iqbal M Rasheed R Hussain I Riaz M Arif MS. Environmental stress and secondary metabolites in plants: an overview. In: Plant Metabolites and Regulation Under Environmental Stress. eds. Ahmad P. Ahanger M. A. Singh V. P. Tripathi D. K. Alam P. Alyemeni M. B. T. Cambridge, MA: Academic Press (2018). 153–67.

[7] Gülçin İ Topal F Sarikaya SBÖ Bursal E Bilsel G Gören AC. Polyphenol contents and antioxidant properties of Medlar *Mespilus germanica* L. Rec. Nat. Prod. (2011) 5:158.

[8] Gulcin İ. Antioxidants and antioxidant methods: an updated overview. Arch Toxicol. (2020) 94:651–715. doi: 10.1007/s00204-020-02689-3 32180036

[9] Waterman PG Mole S. Extrinsic factors influencing production of secondary metabolites in plants. In: Insect-plant Interactions. Boca Raton, FL: CRC press (2019). 107–34.

[10] Olivoto T Nardino M Carvalho IR Follmann DN Szareski VIJ Ferrari M. Plant secondary metabolites and its dynamical systems of induction in response to environmental factors: a review. Afr J Agric Res. (2017) 12:71–84. doi: 10.5897/AJAR2016.11677

[11] Pagare S Bhatia M Tripathi N Pagare S Bansal YK. Secondary metabolites of plants and their role: overview. Curr. Trends Biotechnol. Pharm. (2015) 9:293–304.

[12] Santhi K Sengottuvel R. Qualitative and quantitative phytochemical analysis of *Moringa concanensis* Nimmo. Int J Curr Microbiol App Sci. (2016) 5:633–40. doi: 10.20546/ijcmas.2016.501.064

[13] Shalaby EA Mahmoud GI Shanab SM. Suggested mechanism for the effect of sweeteners on radical scavenging activity of phenolic compounds in black and green tea. Front. Life Sci. (2016) 9:241–51. doi: 10.1080/21553769.2016.1233909

[14] Skrypnik L Grigorev N Michailov D Antipina M Danilova M Pungin A. Comparative study on radical scavenging activity and phenolic compounds content in water bark extracts of alder (*Alnus glutinosa* (L.) Gaertn.), oak (*Quercus robur* L.) and pine (*Pinus sylvestris* L.). Eur. J. Wood Wood Prod. (2019) 77:879–90. doi: 10.1007/s00107-019-01446-3

[15] Alghamdi HA. A need to combat COVID-19; herbal disinfection techniques, formulations and preparations of human health friendly hand sanitizers. Saudi J. Biol. Sci. (2021) 28:3943–7. doi: 10.1016/j.sjbs.2021.03.077, PMID: 33850423 PMC8032473

[16] Biluca FC da Silva B Caon T Mohr ETB Vieira GN Gonzaga LV. Investigation of phenolic compounds, antioxidant and anti-inflammatory activities in stingless bee honey (Meliponinae). Food Res Int. (2020) 129:108756. doi: 10.1016/j.foodres.2019.108756, PMID: 32036884

[17] Ruiz-Ruiz JC Matus-Basto AJ Acereto-Escoffié P Segura-Campos MR. Antioxidant and anti-inflammatory activities of phenolic compounds isolated from *Melipona beecheii* honey. Food Agric Immunol. (2017) 28:1424–37. doi: 10.1080/09540105.2017.1347148

[18] Thakur S Kaurav H Chaudhary G. *Terminalia arjuna*: a potential ayurvedic cardio tonic. Int. J. Res. Appl. Sci. Biotechnol. (2021) 8:227–36. doi: 10.31033/ijrasb.8.2.30

[19] Gioia Di F Petropoulos SA. Phytoestrogens, phytosteroids and saponins in vegetables: biosynthesis, functions, health effects and practical applications. In: Advances in Food and Nutrition Research, vol. 90. eds. Ahmad P. Ahanger M. A. Singh V. P. Tripathi D. K. Alam P. Alyemeni M. B. T. Cambridge, MA: Academic Press (2019). 351–421. 10.1016/bs.afnr.2019.02.004 31445599

[20] Messaoudi O Gouzi H El-Hoshoudy AN Benaceur F Patel C Goswami D. Berries anthocyanins as potential SARS-CoV–2 inhibitors targeting the viral attachment and replication; molecular docking simulation. Egypt J Pet. (2021) 30:33–43. doi: 10.1016/j.ejpe.2021.01.001

[21] Mohammadi Pour P Fakhri S Asgary S Farzaei MH Echeverria J. The signaling pathways, and therapeutic targets of antiviral agents: focusing on the antiviral approaches and clinical perspectives of anthocyanins in the management of viral diseases. Front Pharmacol. (2019) 10:1207. doi: 10.3389/fphar.2019.01207, PMID: 31787892 PMC6856223

[22] Rasouli H Farzaei MH Mansouri K Mohammadzadeh S Khodarahmi R. Plant cell cancer: may natural phenolic compounds prevent onset and development of plant cell malignancy? A literature review. Molecules. (2016) 21:1104. doi: 10.3390/molecules21091104, PMID: 27563858 PMC6274315

[23] Akter R Chowdhury MA Rahman MH. Flavonoids and polyphenolic compounds as potential talented agents for the treatment of Alzheimer’s disease and their antioxidant activities. Curr Pharm Des. (2021) 27:345–56. doi: 10.2174/1381612826666201102102810, PMID: 33138754

[24] Nemzer BV Kalita D Yashin AY Yashin YI. Bioactive compounds, antioxidant activities, and health beneficial effects of selected commercial berry fruits: a review. J Food Res. (2020) 9:78–101. doi: 10.5539/jfr.v9n5p78

[25] Balaydın HT Gülçin İ Menzek A Göksu S Şahin E. Synthesis and antioxidant properties of diphenylmethane derivative bromophenols including a natural product. J Enzyme Inhib Med Chem. (2010) 25:685–95. doi: 10.3109/14756360903514164, PMID: 20113195

[26] Elmastas M Turkekul I Ozturk L Gulcin I Isildak O Aboul-Enein HY. Antioxidant activity of two wild edible mushrooms (Morchella vulgaris and Morchella esculanta) from North Turkey. Comb Chem High Throughput Screen. (2006) 9:443–8. doi: 10.2174/138620706777698544, PMID: 16842225

[27] Gülçin I. Measurement of antioxidant ability of melatonin and serotonin by the DMPD and CUPRAC methods as trolox equivalent. J Enzyme Inhib Med Chem. (2008) 23:871–6. doi: 10.1080/14756360701626223 18608760

[28] Alkadi H. A review on free radicals and antioxidants. Inf Disord-Drug Targets (Formerly Current Drug Targets-Infectious Disorders). (2020) 20:16–26. 10.2174/1871526518666180628124323 29952268

[29] Brand MD. Mitochondrial generation of superoxide and hydrogen peroxide as the source of mitochondrial redox signaling. Free Radic Biol Med. (2016) 100:14–31. doi: 10.1016/j.freeradbiomed.2016.04.001 27085844

[30] Ishihara G Kawamoto K Komori N Ishibashi T. Molecular hydrogen suppresses superoxide generation in the mitochondrial complex I and reduced mitochondrial membrane potential. Biochem Biophys Res Commun. (2020) 522:965–70. doi: 10.1016/j.bbrc.2019.11.135, PMID: 31810604

[31] Poljšak B Fink R. The protective role of antioxidants in the Defence against ROS/RNS-mediated environmental pollution. Oxidative Med Cell Longev. (2014) 2014:1–22. doi: 10.1155/2014/671539, PMID: 25140198 PMC4129148

[32] Shadyro O Samovich S Edimecheva I Novitsky R Khrutskin V Ihnatovich L. Potential role of free-radical processes in biomolecules damage during COVID-19 and ways of their regulation. Free Radic Res. (2021) 55:665–76. doi: 10.1080/10715762.2021.1938024, PMID: 34085882

[33] Tvrdá E Benko F. Free radicals: what they are and what they do. In: Pathology. Cambridge, MA: Academic Press (2020). 3–13.

[34] Chuang GC Xia H Mahne SE Varner KJ. Environmentally persistent free radicals cause apoptosis in HL-1 cardiomyocytes. Cardiovasc Toxicol. (2017) 17:140–9. doi: 10.1007/s12012-016-9367-x, PMID: 27052339 PMC5053829

[35] Liguori I Russo G Curcio F Bulli G Aran L Della-Morte D. Oxidative stress, aging, and diseases. Clin Interv Aging. (2018) 13:757–72. doi: 10.2147/CIA.S158513, PMID: 29731617 PMC5927356

[36] Luo J Mills K le Cessie S Noordam R van Heemst D. Ageing, age-related diseases and oxidative stress: what to do next? Ageing Res Rev. (2020) 57:100982. doi: 10.1016/j.arr.2019.100982, PMID: 31733333

[37] Magnani F Mattevi A. Structure and mechanisms of ROS generation by NADPH oxidases. Curr Opin Struct Biol. (2019) 59:91–7. doi: 10.1016/j.sbi.2019.03.001, PMID: 31051297

[38] Pizzino G Irrera N Cucinotta M Pallio G Mannino F Arcoraci V. Oxidative stress: harms and benefits for human health. Oxidative Med Cell Longev. (2017) 2017:1–13. doi: 10.1155/2017/8416763, PMID: 28819546 PMC5551541

[39] Zhang J Wang X Vikash V Ye Q Wu D Liu Y. ROS and ROS-mediated cellular signaling. Oxidative Med Cell Longev. (2016) 2016:1–18. doi: 10.1155/2016/4350965, PMID: 26998193 PMC4779832

[40] Nyero A Achaye I Odongo W Anywar G Malinga GM. Wild and semi-wild edible plants used by the communities of Acholi sub-region, northern Uganda. Ethnobot Res Appl. (2021) 21:1–12. doi: 10.32859/era.21.16.1-12

[41] Oryema C Oryem-Origa H. Analysis of the fresh pulps of *Borassus aethiopum* fruits of Gulu district, Uganda. Am J Food Nutr. (2016) 4:177–18.

[42] WHO. WHO Guidelines on Good Agricultural and Collection Practices (GACP) for Medicinal Plants. Geneva: World Health Organization (2004).

[43] Goode PM. Edible Plants of Uganda: The Value of Wild and Cultivated Plants as Food, vol. 41. Rome: Food & Agriculture Organization (1989).

[44] Gebremeskel AF Ngoda PN Kamau-Mbuthia EW Mahungu SM. The effect of roasting, storage temperature, and ethanoic basil (*Ocimum basilicum* L.) extract on the oxidative stability of crude sesame (*Sesamum indicum* L.) oil. Food Science & Nutrition. Food Sci Nutr. (2022) 10:2736–48. doi: 10.1002/fsn3.2877, PMID: 35959257 PMC9361459

[45] El-Hadary AE Taha M. Pomegranate peel methanolic-extract improves the shelf-life of edible-oils under accelerated oxidation conditions. Food Sci Nutr. (2020) 8:1798–811. doi: 10.1002/fsn3.1391, PMID: 32328245 PMC7174205

[46] Culei I. Methodology for Analysis of Vegetable Drugs. Practical Manuals of the Industrial Utilization of Medical and Aromatic Plants. Bucharest: Bucharest Office of Joint UNIDO (1989).

[47] Evans WC. Trease and Evans' Pharmacognosy. 16th ed. Edinburgh: Elsevier (2009).

[48] Sarker SD Nahar L. An introduction to natural products isolation. Nat Products Isolation. (2012) 864:1–25. doi: 10.1007/978-1-61779-624-1_1 22367891

[49] Köksal E Bursal E Gülçin İ Korkmaz M Çağlayan C Gören AC. Antioxidant activity and polyphenol content of Turkish thyme (*Thymus vulgaris*) monitored by liquid chromatography and tandem mass spectrometry. Int J Food Prop. (2017) 20:514–25. doi: 10.1080/10942912.2016.1168438

[50] Makkar HP. Measurement of total phenolics and tannins using Folin-Ciocalteu method. In: Quantification of Tannins in Tree and Shrub Foliage. Dordrecht: Springer (2003). 49–51.

[51] Zeb A. Phenolic profile and antioxidant potential of wild watercress (*Nasturtium officinale* L.). Springer Plus. (2015) 4:1–7. doi: 10.1186/s40064-015-1514-5 26636002 PMC4656250

[52] Khan AS Arif K Munir B Kiran S Jalal F Qureshi N. Estimating total phenolics in Taraxacum officinale (L.) extracts. Pol J Environ Stud. (2019) 28:497:–501. doi: 10.15244/pjoes/78435

[53] Ordonez AAL Gomez JD Vattuone MA. Antioxidant activities of Sechium edule (Jacq.) Swartz extracts. Food Chem. (2006) 97:452–8. doi: 10.1016/j.foodchem.2005.05.024

[54] Harborne AJ. Phytochemical methods a guide to modern techniques of plant analysis. Springer Science & Business Media. (1998).

[55] Obadoni BO Ochuko PO. Phytochemical studies and comparative efficacy of the crude extracts of some haemostatic plants in Edo and Delta states of Nigeria. Global J Pure Appl Sci. (2002) 8:203–8. doi: 10.4314/gjpas.v8i2.16033

[56] Abdul-Wahab NZ Shahar S Abdullah-Sani H Pihie AHL Ibrahim N. Antioxidant, antibacterial and antiviral properties of *Goniothalamus umbrosus* leaves methanolic extract. Afr J Microbiol Res. (2011) 5:3138–43. doi: 10.5897/AJMR10.758

[57] Senguttuvan J Paulsamy S Karthika K. Phytochemical analysis and evaluation of leaf and root parts of the medicinal herb, *Hypochaeris radicata* L for in vitro antioxidant activities. Asian Pacific J. Trop. Biomed. (2014) 4:S359–67. doi: 10.12980/APJTB.4.2014C1030, PMID: 25183112 PMC4025295

[58] Emmanuel TV Njoka JT Catherine LW Lyaruu HVM. Nutritive and anti-nutritive qualities of mostly preferred edible woody plants in selected drylands of Iringa District, Tanzania. Pak J Nutr. (2011) 10:786–91. doi: 10.3923/pjn.2011.786.791

[59] Karimi A Krähmer A Herwig N Schulz H Hadian J Meiners T. Variation of secondary metabolite profile of Zataria multiflora Boiss. Populations linked to geographic, climatic, and edaphic factors. Front Plant Sci. (2020) 11:969. doi: 10.3389/fpls.2020.00969, PMID: 32719699 PMC7348666

[60] Wen B Ren S Zhang Y Duan Y Shen J Zhu X. Effects of geographic locations and topographical factors on secondary metabolites distribution in green tea at a regional scale. Food Control. (2020) 110:106979. doi: 10.1016/j.foodcont.2019.106979

[61] Hassan ZM Manyelo TG Selaledi L Mabelebele M. The effects of tannins in monogastric animals with special reference to alternative feed ingredients. Molecules. (2020) 25:4680. doi: 10.3390/molecules25204680, PMID: 33066367 PMC7587385

[62] Toma L Sanda GM Niculescu LS Deleanu M Sima AV Stancu CS. Phenolic compounds exerting lipid-regulatory, anti-inflammatory and epigenetic effects as complementary treatments in cardiovascular diseases. Biomol Ther. (2020) 10:641. doi: 10.3390/biom10040641, PMID: 32326376 PMC7226566

[63] Farha AK Yang QQ Kim G Li HB Zhu F Liu HY. Tannins as an alternative to antibiotics. Food Biosci. (2020) 38:100751. doi: 10.1016/j.fbio.2020.100751

[64] Baer-Dubowska W Szaefer H Majchrzak-Celińska A Krajka-Kuźniak V. Tannic acid: specific form of tannins in cancer chemoprevention and therapy-old and new applications. Curr Pharmacol Rep. (2020) 6:28–37. doi: 10.1007/s40495-020-00211-y

[65] Rajasekar N Sivanantham A Ravikumar V Rajasekaran S. An overview on the role of plant-derived tannins for the treatment of lung cancer. Phytochemistry. (2021) 188:112799. doi: 10.1016/j.phytochem.2021.112799, PMID: 33975161

[66] Andabati B Muyonga J. Phenolic content and antioxidant activity of selected Ugandan traditional medicinal foods. Afr J Food Sci. (2014) 8:427–34. doi: 10.5897/AJFS2014.1136

[67] Lamien-Meda A Lamien CE Compaoré MM Meda RN Kiendrebeogo M Zeba B. Polyphenol content and antioxidant activity of fourteen wild edible fruits from Burkina Faso. Molecules. (2008) 13:581–94. doi: 10.3390/molecules13030581, PMID: 18463567 PMC6245336

[68] Yang RY Lin S Kuo G. Content and distribution of flavonoids among 91 edible plant species. Asia Pacific journal of clinical nutrition. (2008) 17:275–279. 18296355

[69] Imran M Rauf A Shah ZA Saeed F Imran A Arshad MU. Chemo-preventive and therapeutic effect of the dietary flavonoid kaempferol: a comprehensive review. Phytother Res. (2019) 33:263–75. doi: 10.1002/ptr.6227, PMID: 30402931

[70] Ullah A Munir S Badshah SL Khan N Ghani L Poulson BG. Important flavonoids and their role as a therapeutic agent. Molecules. (2020) 25:5243. doi: 10.3390/molecules25225243, PMID: 33187049 PMC7697716

[71] Jia JY Zang EH Lv LJ Li QY Zhang CH Xia Y. Flavonoids in myocardial ischemia-reperfusion injury: therapeutic effects and mechanisms. Chin Herbal Med. (2021) 13:49–63. doi: 10.1016/j.chmed.2020.09.002, PMID: 36117755 PMC9476686

[72] Nakayama T Takahashi S Waki T. Formation of flavonoid metabolons: functional significance of protein-protein interactions and impact on flavonoid chemodiversity. Front Plant Sci. (2019) 10:821. doi: 10.3389/fpls.2019.00821, PMID: 31338097 PMC6629762

[73] Bezruk I Materiienko A Gubar S Proskurina K Budanova L Ivanauskas L. Estimation of the influence of the environmental factors on the accumulation of phytochemicals and antioxidant capacity in the ivy leaves (*Hedera helix* L.). Nat Prod Res. (2022) 36:1014–9. doi: 10.1080/14786419.2020.1843029, PMID: 33146030

[74] Nguyen KQ Scarlett CJ Vuong QV. Assessment and comparison of phytochemicals and antioxidant properties from various parts of the Australian maroon bush (*Scaevola spinescens*). Heliyon. (2021) 7:e06810. doi: 10.1016/j.heliyon.2021.e06810, PMID: 33981883 PMC8082193

[75] Nawaz H Shad MA Rehman N Andaleeb H Ullah N. Effect of solvent polarity on extraction yield and antioxidant properties of phytochemicals from bean (*Phaseolus vulgaris*) seeds. Braz J Pharm Sci. (2020) 56:1–9. doi: 10.1590/s2175-97902019000417129

[76] Orech FO Akenga T Ochora J Friis H Aagaard-Hansen J. Potential toxicity of some traditional leafy vegetables consumed in Nyang’oma division, Western Kenya. Afr J Food Agric Nutr Dev. (2005) 5:1–14.

[77] Sun Q Fu Q Li S Li J Liu S Wang Z. Emetine exhibits anticancer activity in breast cancer cells as an antagonist of Wnt/β-catenin signaling. Oncol Rep. (2019) 42:1735–44. doi: 10.3892/or.2019.7290, PMID: 31436297 PMC6775799

[78] Uzor PF. Recent developments on potential new applications of emetine as anti-cancer agent. EXCLI J. (2016) 15:323. 27366142 10.17179/excli2016-280 PMC4928012

[79] Jadid N. Hidayati D. Hartanti S. R. Arraniry B. A. Rachman R. Y. Wikanta W. (2017). Antioxidant activities of different solvent extracts of Piper retrofractum Vahl. Using DPPH assay. In *AIP Conference Proceedings* (Vol. 1854, No. 1, p. 020019). New York, NY: AIP Publishing LLC.

[80] Seal T. Antioxidant activity of some wild edible plants of Meghalaya state of India: a comparison using two solvent extraction systems. Int. J. Nutr. Metab. (2012) 4:51–6.

[81] Do QD Angkawijaya AE Tran-Nguyen PL Huynh LH Soetaredjo FE Ismadji S. Effect of extraction solvent on total phenol content, total flavonoid content, and antioxidant activity of Limnophila aromatica. J Food Drug Anal. (2014) 22:296–302. doi: 10.1016/j.jfda.2013.11.001, PMID: 28911418 PMC9354875

[82] Moo-Huchin VM Canto-Pinto JC Cuevas-Glory LF Sauri-Duch E Pérez-Pacheco E Betancur-Ancona D. Effect of extraction solvent on the phenolic compounds content and antioxidant activity of Ramon nut (*Brosimum alicastrum*). Chem Pap. (2019) 73:1647–57. doi: 10.1007/s11696-019-00716-x

[83] Brighente IMC Dias M Verdi LG Pizzolatti MG. Antioxidant activity and total phenolic content of some Brazilian species. Pharm Biol. (2007) 45:156–61. doi: 10.1080/13880200601113131

[84] Deng B Cao Y Fang S Shang X Yang W Qian C. Variation and stability of growth and leaf flavonoid content in *Cyclocarya paliurus* across environments. Ind Crop Prod. (2015) 76:386–93. doi: 10.1016/j.indcrop.2015.07.011

[85] Khodabande Z Jafarian V Sariri R. Antioxidant activity of *Chelidonium majus* extract at phenological stages. Appl Biol Chem. (2017) 60:497–503. doi: 10.1007/s13765-017-0304-x

[86] Noreen H Semmar N Farman M McCullagh JS. Measurement of total phenolic content and antioxidant activity of aerial parts of medicinal plant *Coronopus didymus*. Asian Pac J Trop Med. (2017) 10:792–801. doi: 10.1016/j.apjtm.2017.07.024, PMID: 28942828

